# Soft‐Tissue‐Focused Orthodontics in Patients With Stage IV Periodontitis: Segmented Arch Technique and Skeletal Anchorage: A Case Report

**DOI:** 10.1002/ccr3.72702

**Published:** 2026-05-27

**Authors:** Lisa‐Marie Mai, Bernhard Wiechens, Anja Quast, Gaayathiri Suntharalingam, Valentina Hrasky, Philipp Meyer‐Marcotty

**Affiliations:** ^1^ Department of Orthodontics University Medical Center Göttingen Göttingen Germany; ^2^ Department of Preventive Dentistry, Periodontology, and Cariology University Medical Center Göttingen Göttingen Germany

**Keywords:** adolescent health, facial soft tissue profile, interdisciplinary communication, lip‐incisor interaction, orthodontics, periodontics

## Abstract

Orthodontic treatment in patients with advanced periodontitis is often considered high‐risk due to impaired periodontal anchorage and increased susceptibility to treatment‐related damage. This report presents a 60‐year‐old woman with stage IV periodontitis, upper anterior protrusion (overjet 7.5 mm), crowding, and advanced vertical bone loss. Treatment with a maxillary fixed appliance and skeletal anchorage over 9 months enabled intrusion and retraction of the anterior teeth, closure of a lateral diastema, and reduction of overjet, achieving functional and aesthetic improvement without compromising periodontal support. Orthodontic treatment is achievable and effective in patients in later adulthood with advanced periodontitis. Using only small, segmented appliances with a controlled force vector guided by skeletal anchorage allows controlled and secure anterior tooth movement. Considering the incisor/lip interaction according to the FAOP enables reliable tooth movements, improving function, aesthetics, and long‐term tooth preservation.

## Key Clinical Message

In patients with stage IV periodontitis, orthodontic treatment is feasible after successful periodontal stabilization. Close collaboration between periodontist and orthodontist, combined with controlled biomechanics, is essential. Segmented orthodontic mechanics with skeletal anchorage allow safe and controlled intrusion and retrusion of elongated incisors while preserving compromised periodontal support.

## Graphical abstract

Correction of Soft Tissue Profile Disortion Through Periodontal‐Orthodontic Treatment: Advanced periodontitis with pathologic anterior tooth migration resulted in protrusion and distortion of the soft tissue profile. Following periodontal stabilization, segmented orthodontic mechanics with skeletal anchorage enabled controlled intrusion and retrusion of the maxillary incisors, improving facial profile, function, and aesthetics while preserving compromised periodontal support.

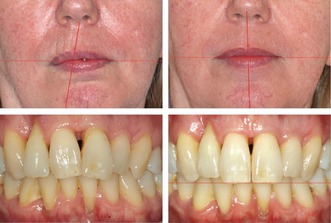

**Abbreviations**
EFP: European Federation of Periodontology
FAOP: Functional Aesthetic Occlusal Plane

## 1 Introduction

**FIGURE 1:**
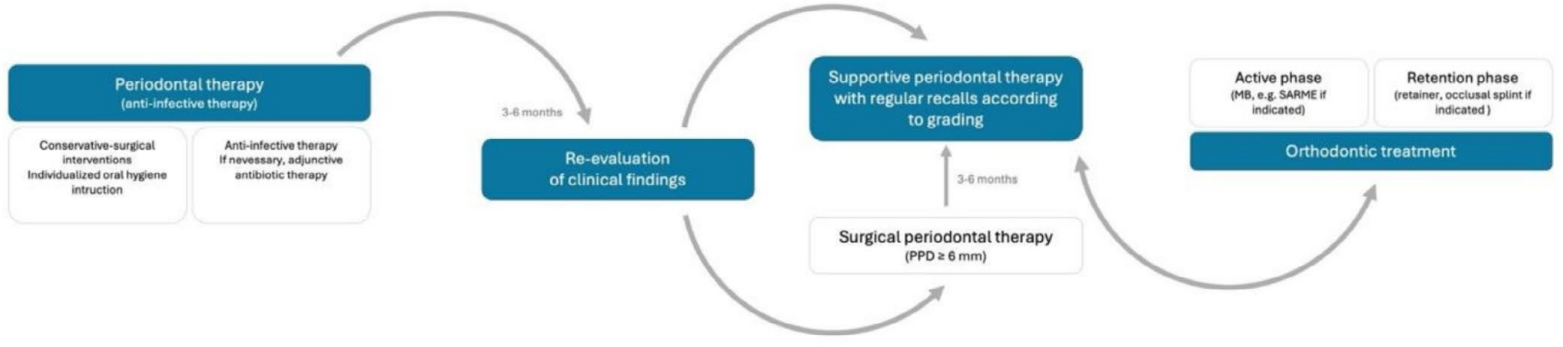
Chronological overview of the treatment sequence including initial periodontal therapy, orthodontic therapy and the final retention phase. MB, multibracket appliance; PPD, probing pocket depths; SARME, surgically assisted rapid maxillary expansion. The figure was adapted from Herrera et al. [6], treatment of stage IV periodontitis: The EFP S3 guideline.

The number of orthodontic treatments in adults has increased significantly over the past 20 years, which can be attributed to a growing awareness of functional improvements and oral health [1]. Malocclusion in adults is often associated with underlying periodontitis, frequently leading to characteristic changes such as proclination and overeruption of the anterior teeth [2]. Periodontitis is particularly widespread among older adults, with more than 50% of patients over 65 affected by severe periodontitis (stage III and IV) [3] and can lead to pathological tooth migration in more than half of those affected [4, 5]. Such cases require treatment strategies that address the reduced periodontal support in order to achieve stable outcomes (Figure 1).

Oral health is understood as a complex condition that encompasses disease status, psychosocial well‐being, as well as physiological function [7]. Although a direct link between the correction of malocclusions and the progression of diseases has not yet been clearly established [8], orthodontic treatments have been shown to have positive effects on speech, masticatory function, and psychosocial quality of life [6, 9, 10]. The etiology of malocclusion is multifactorial [11, 12, 13, 14], with periodontitis leading to pathological tooth migration due to loss of connective tissue attachment and bone resorption [4, 15, 16, 17].

These tooth migrations can lead to functional and aesthetic impairments that negatively affect mental health including self‐esteem and social interactions [5, 17, 18]. As a central means of nonverbal communication, an impaired smile can increase psychosocial stress [19, 20]. Severe malocclusion has been shown to correlate more strongly with psychological distress than other aesthetic parameters [21, 22].

**FIGURE 2:**
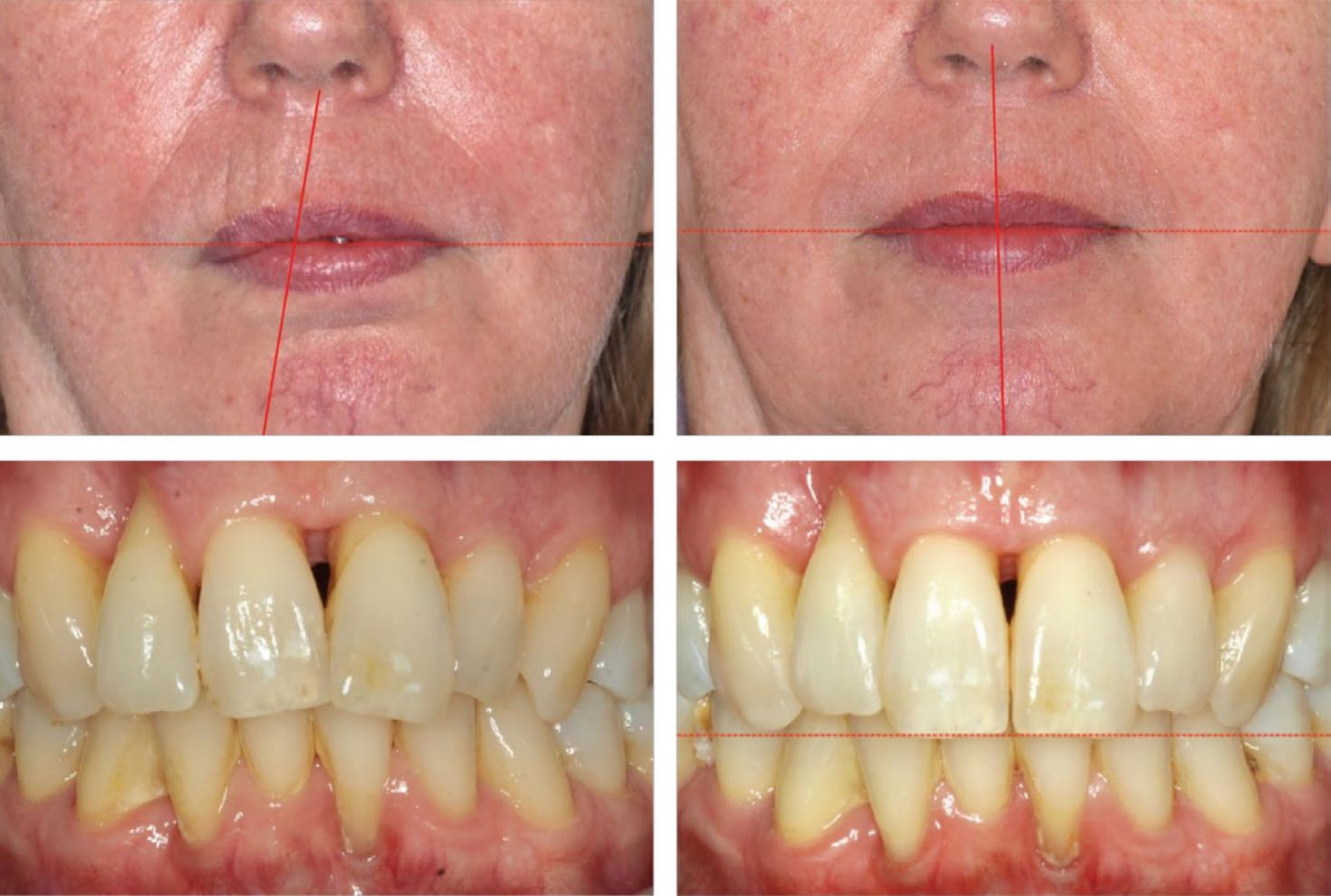
Effect of tooth position on the soft tissue profile. Left: pre‐treatment, right: post‐treatment. Upper row: extraoral views; lower row: intraoral views.

In addition to these psychosocial effects, malocclusion can cause measurable changes in the facial soft tissue profile. Studies have shown that the vertical and sagittal relationships of the anterior teeth play a role in shaping the soft tissue contours, particularly influencing the upper lip [23, 24, 25, 26]. Pathological tooth migration, including a deep overbite or anterior proclination, can lead to visible alterations, negatively impacting facial harmony (Figure 2).

**FIGURE 3:**
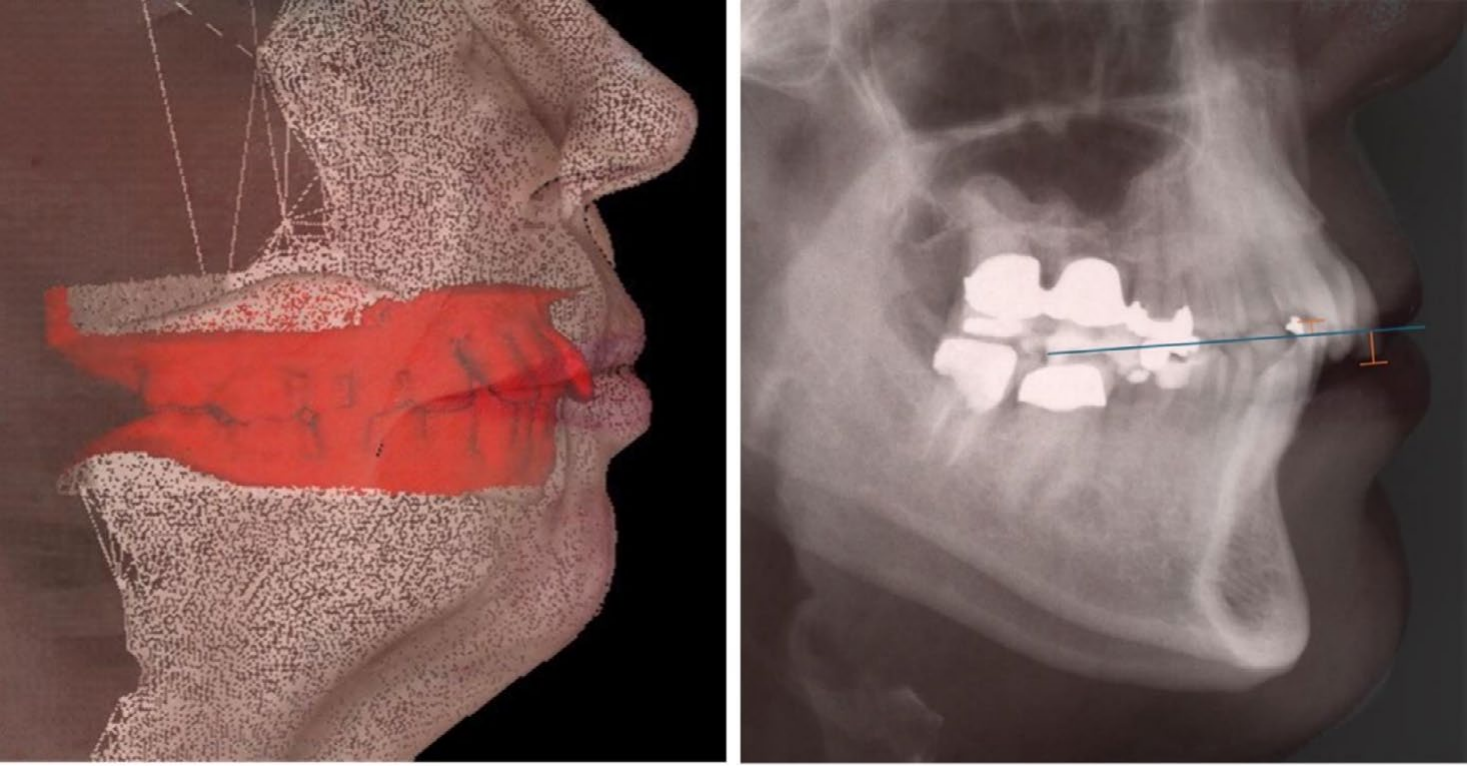
Combined visualization of occlusal plane and facial structures. Left: 3D visualization of tooth position and soft‐tissue contours, created by superimposing a 3D facial photograph with a dental scan (red). Right: The Functional‐Aesthetic‐Occlusal Plane (FAOP) visualized on a cephalometric radiograph, overlaid with an extraoral photograph for illustration. The blue line represents the FAOP, defined from the contact point of the maxillary and mandibular first molars to the upper lip stomion. The orange lines indicate the distances of the maxillary and mandibular incisors to the FAOP; in this case, the incisors are positioned clearly above the normative range.

Clinical imaging provides valuable insights into the relationship between dental structures and soft tissues. Three‐dimensional facial photographs combined with dental scans enable precise visualization of the soft tissue profile as well as the position of the teeth (Figure 3). Such imaging techniques illustrate how malocclusion can influence lip posture and perioral balance, with orthodontic correction helping to achieve a more balanced soft tissue appearance.

Although orthodontic interventions can significantly improve treatment outcomes and quality of life in patients with periodontal disease [17, 27], they are often insufficiently considered in clinical communication [5]. The current S3 guideline of the European Federation of Periodontology (EFP) recognizes orthodontic therapy for stage IV periodontitis as an integral part of interdisciplinary treatment—provided that periodontal stability has been achieved beforehand [6] (Figure 1).

Classification of periodontitis is based on stages and grades. The stages provide information on the severity, complexity, extent, and distribution of the disease. Stage IV represents the most severe form of periodontitis with very high attachment loss (≥ 8 mm), pathological tooth migration, bone loss extending to the apical third of the roots, tooth loss (≥ 5 teeth), and complex clinical features such as tooth mobility [28].

According to the EFP S3 level clinical practice guideline [6], patients in Stage IV typically require an interdisciplinary therapeutic regimen, including periodontal‐orthodontic sequences, for complex rehabilitation to restore function and aesthetics. The grades, on the other hand, reflect biological characteristics such as disease progression rate, response to therapy, and systemic impact, with a primary criterion being evidence of progression, assessed directly or indirectly by bone loss relative to the patient's age [28].

The use of low and precisely controlled forces is essential to accommodate the compromised periodontal condition [1, 29]. Controlled tooth movement is achieved through segmented multibracket appliances and skeletal anchorage systems [30, 31], while a carefully controlled retention phase is considered essential [32].

A key objective in these cases is the retrusion and intrusion of the anterior teeth to re‐establish a functional and aesthetically balanced position [33, 34, 35]. Consideration should also be given to the Functional Aesthetic Occlusal Plane (FAOP), which is described as a reference for the ideal position of the incisal edges and for harmonizing the occlusal plane [36]. As previously mentioned, these corrections can have measurable effects not only on the facial profile but also on lip morphology, influencing both function and aesthetics [37, 38, 39]. The FAOP is defined by a line connecting upper lip stomion with the contact point between the maxillary and mandibular first molars. In this process, the incisal edge of the maxillary incisors should be set 2–4 mm below this plane, whereas the mandibular incisal edges should be precisely aligned with it. These measurements provide a basis for evaluating the functional and aesthetic relationships of the anterior teeth [36].

The following case was selected to illustrate the feasibility and functional benefits of orthodontic treatment in patients in late adulthood with stage IV periodontitis, a population in which data on interdisciplinary orthodontic interventions remain limited.

## 2 Case Report

### 2.1 Diagnosis

#### 2.1.1 Clinical Inspection

**FIGURE 4:**
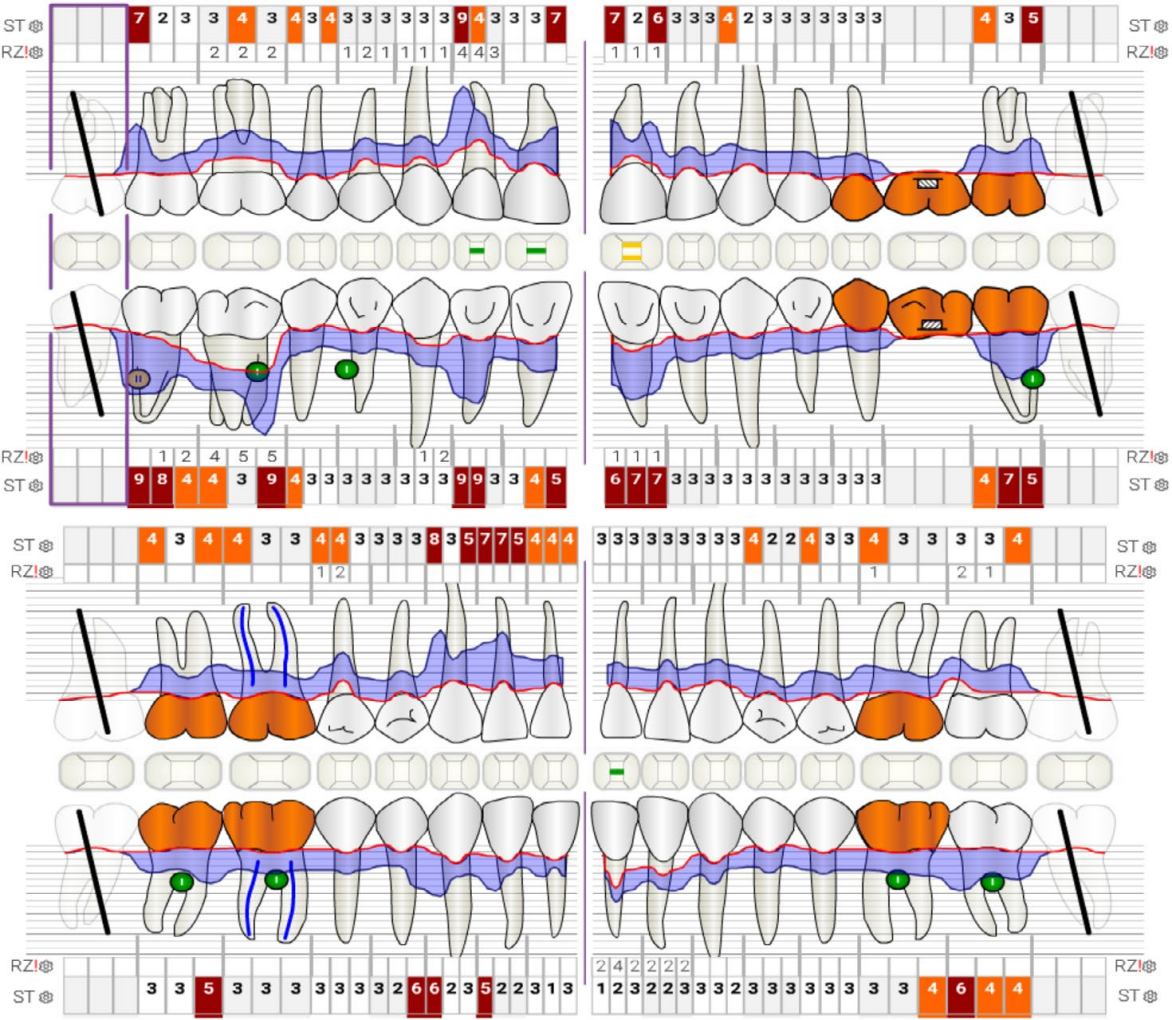
Periodontal status before therapy. Periodontal status before therapy, with RZ indicating gingival recessions and ST indicating probing depths in millimeters.

A 60‐year‐old female patient presented for orthodontic consultation, her chief complaint being the increasing protrusion and elongation of her upper front teeth, which led to habitual lip entrapment with an incompetent lip seal. The patient had a history of chronic periodontitis (stage IV, grade C) with advanced vertical bone loss in the maxillary anterior region and was under regular periodontal maintenance following previous therapy. Clinical periodontal examination revealed generalized increased probing depths, with values of up to 9 mm (Figure 4). She also reported nighttime bruxism, which was managed with an occlusal splint.

**FIGURE 5:**
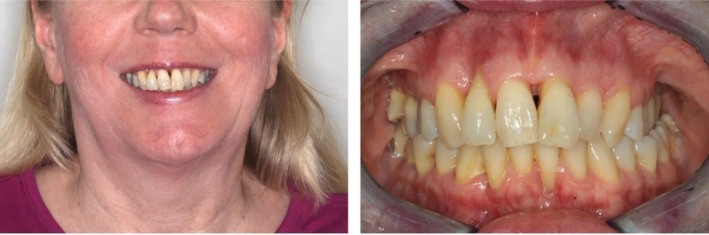
Initial situation.

**FIGURE 6:**
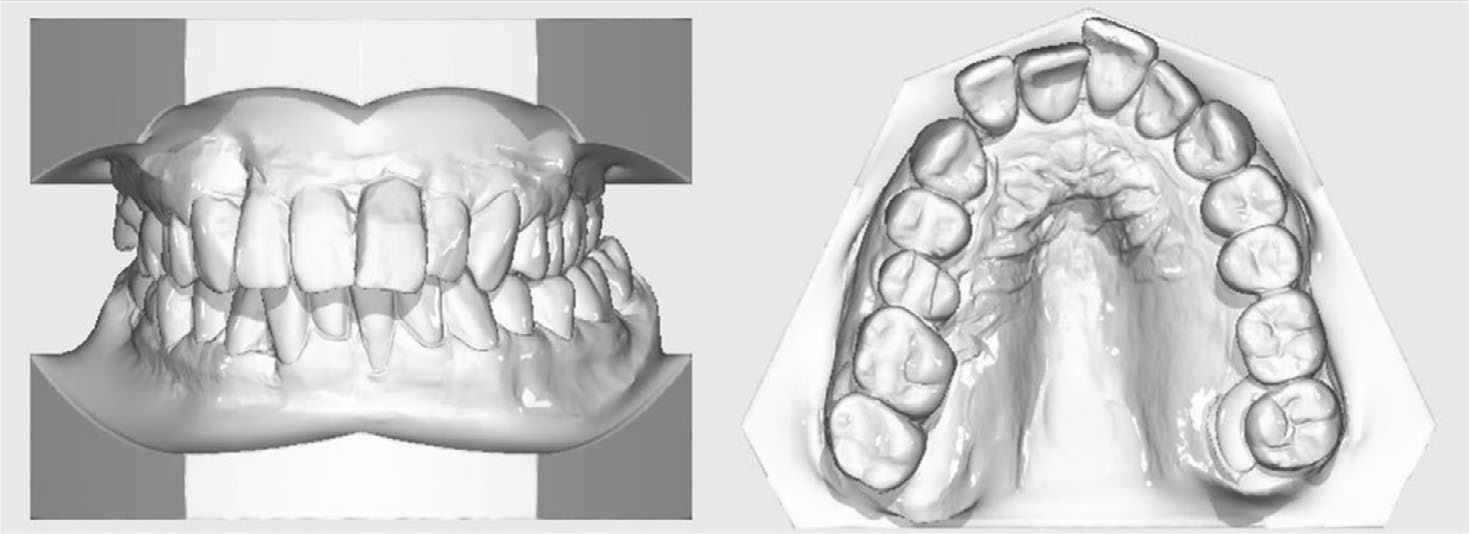
Initial dental models.

Clinical examination revealed a bilateral Angle Class II/1 malocclusion (½ premolar width distal occlusion) with a 7.5 mm overjet and 2 mm overbite. A transverse crossbite was present in regions 15 and from 25 to 27. The upper arch was transversely narrow and featured a steeply inclined and retruded anterior segment with a high palatal vault. Furthermore, labial tipping of tooth 21, supraposition of teeth 11–21, and a lateral diastema distal to tooth 12, as well as anterior crowding, were observed. Functional assessment confirmed symmetrical mouth opening and no restriction in mandibular movements (Figures 5 and 6).

#### 2.1.2 Radiographics

**FIGURE 7:**
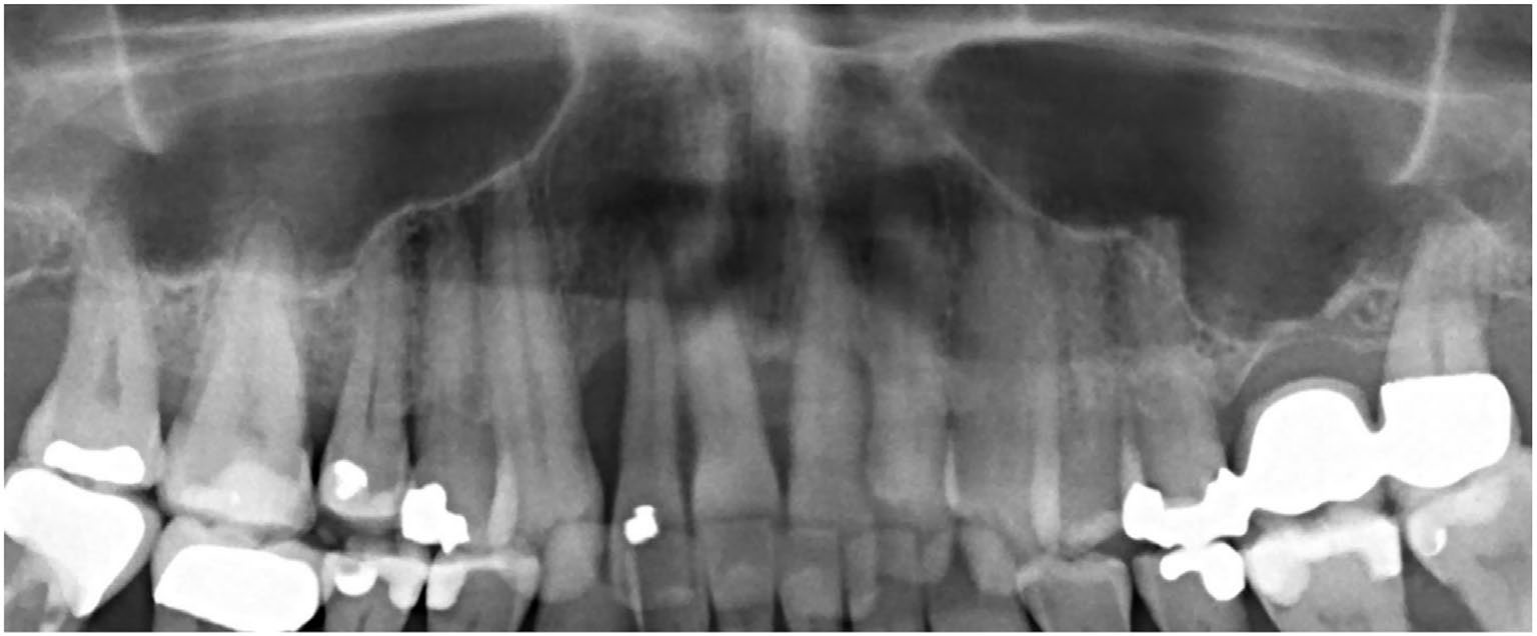
Initial panoramic radiograph showing the maxillary section of the orthopantomogram (OPG).

**FIGURE 8:**
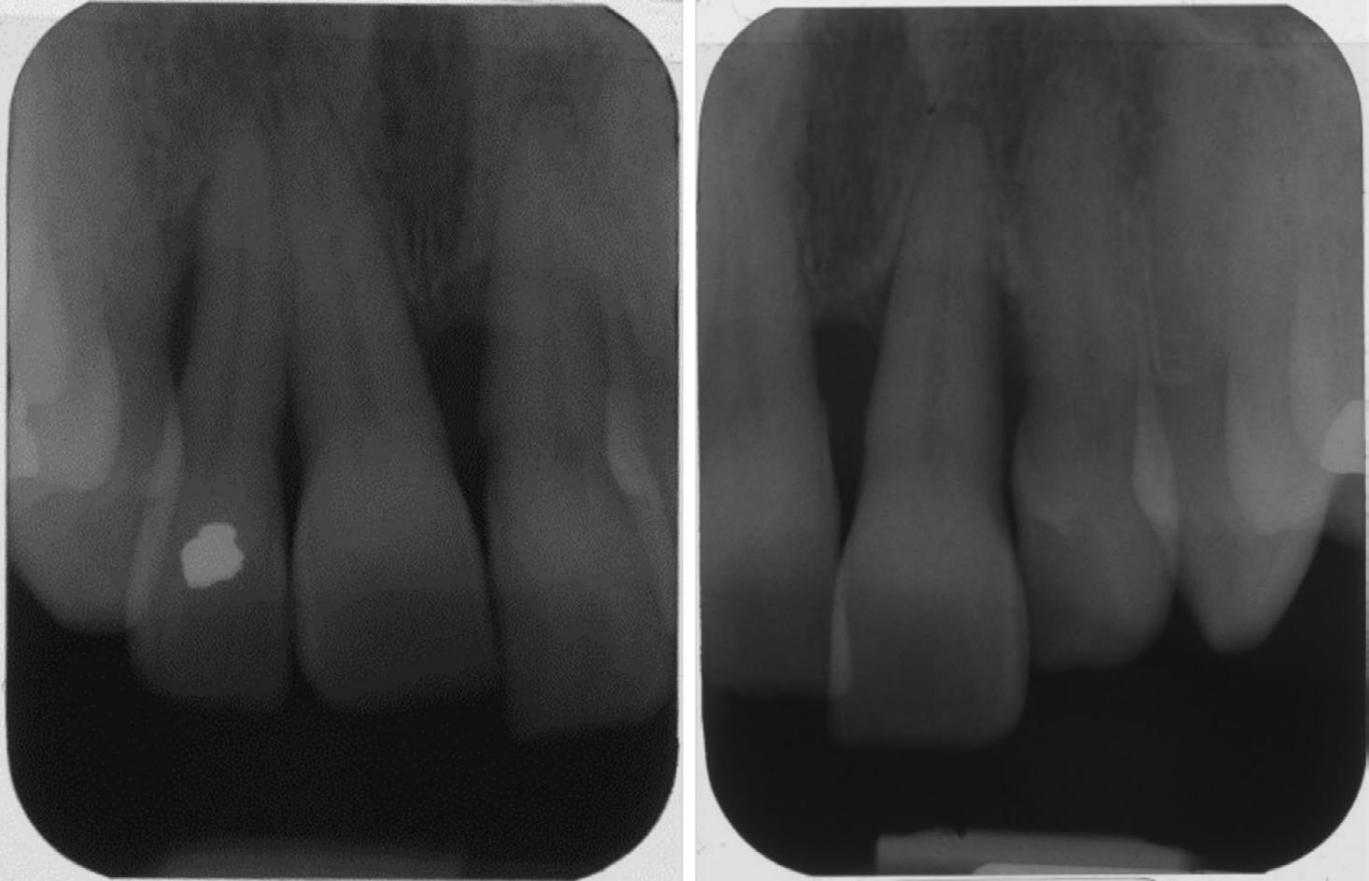
Periapical radiograph of the maxillary anterior tooth showing advanced bone loss.

Radiographic analysis showed a slight hypoplasia of the left condyle, 27 permanent teeth with absence of teeth 18 and 16, and a deep sinus floor in region 26. Generalized vertical and horizontal bone loss was most pronounced in the upper anterior region and around teeth 12 and 22. A bony gap was revealed distal to tooth 12 (Figures 7 and 8).

Cephalometric analysis confirmed retruded upper and protruded lower incisors (U1‐NSL 97.7°, L1‐ML 93.4°).

### 2.2 Etiology

The primary etiology of the malocclusion was pathological tooth migration because of the periodontal destruction. Contributing factors included the skeletal Class II pattern with mandibular retrognathia, the vertical growth tendency as well as the long‐standing imbalance in anterior occlusal contacts. The elongation of the anterior maxillary teeth and lip entrapment (Figure 3) further intensified the functional and aesthetic issues. The patient was a non‐smoker and did not report diabetes or other relevant systemic inflammatory conditions. Arterial hypertension was present but is considered only a minor or inconsistent risk factor for periodontal destruction [40]. The periodontal breakdown was therefore most likely associated with long‐standing biofilm accumulation and individual susceptibility as well as the age of the patient [16, 41, 42].

Apart from the lip entrapment, no additional functional disturbances such as mouth breathing were detected.

### 2.3 Periodontal Therapy and Timing of Orthodontic Treatment

Prior to the initiation of orthodontic treatment, comprehensive periodontal therapy was carried out to control the active inflammatory process and to stabilize the periodontal condition. Microbiological analysis revealed a clinically relevant bacterial load consisting of pathogens associated with the red complex ( *Tannerella forsythia* ) and the orange complex ( *Prevotella intermedia* , *Peptostreptococcus micros* ).

According to the detected bacterial constellation, adjunctive systemic antibiotic therapy was indicated in addition to mechanical periodontal treatment. Therefore, following scaling and root planing, the patient received systemic antibiotic therapy with metronidazole (3 × 400 mg/day for 7 days).

Orthodontic treatment was initiated only after periodontal stabilization had been achieved and the patient had entered a regular periodontal maintenance program.

### 2.4 Treatment Objectives

The main treatment objective was to restore functional anterior guidance and lip competence while preserving the remaining periodontal support. Specifically, the goals were to retract and intrude the elongated teeth, correct the anterior crowding, and close the lateral diastema. Skeletal anchorage was planned to minimize reactive forces on the posterior teeth. An additional aim was to reduce the overjet and overbite while maintaining the existing occlusion in the posterior segments and bite relationship. Final stabilization was planned with a bonded retainer.

### 2.5 Alternatives

A combined orthodontic‐surgical approach was considered including surgically assisted rapid palatal expansion (SARPE) to increase the transverse width of the maxilla. This would have been followed by maxillary multibracket treatment, initially with passive stainless‐steel sectional archwires in the posterior segments. The anterior segment would have been treated separately using a NiTi archwire as well as auxiliary intrusion springs, while a mandibular multibracket appliance was also planned.

Due to the patient's preference for a minimally invasive approach, the combined surgical treatment was not pursued.

### 2.6 Progress

**FIGURE 9:**
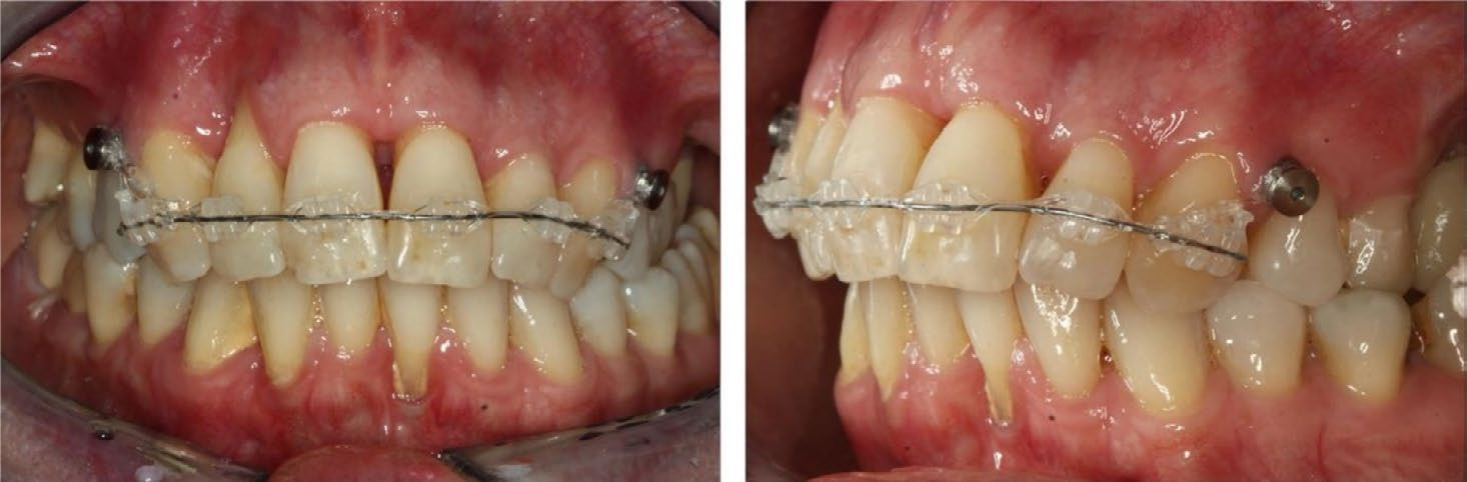
Start of multibracket treatment with sectional arch 13–23 and mini screws.

**FIGURE 10:**
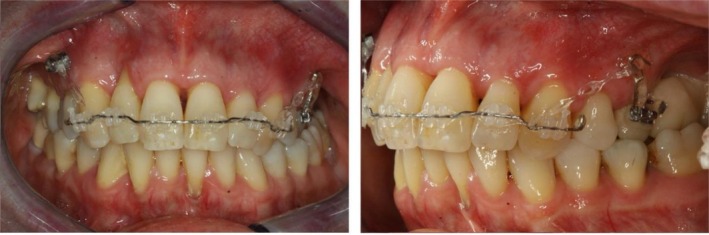
Bonding of tooth 25 with individualized loop.

**FIGURE 11:**
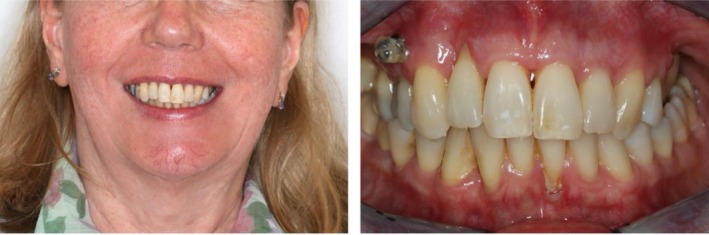
Final treatment outcome.

**FIGURE 12:**
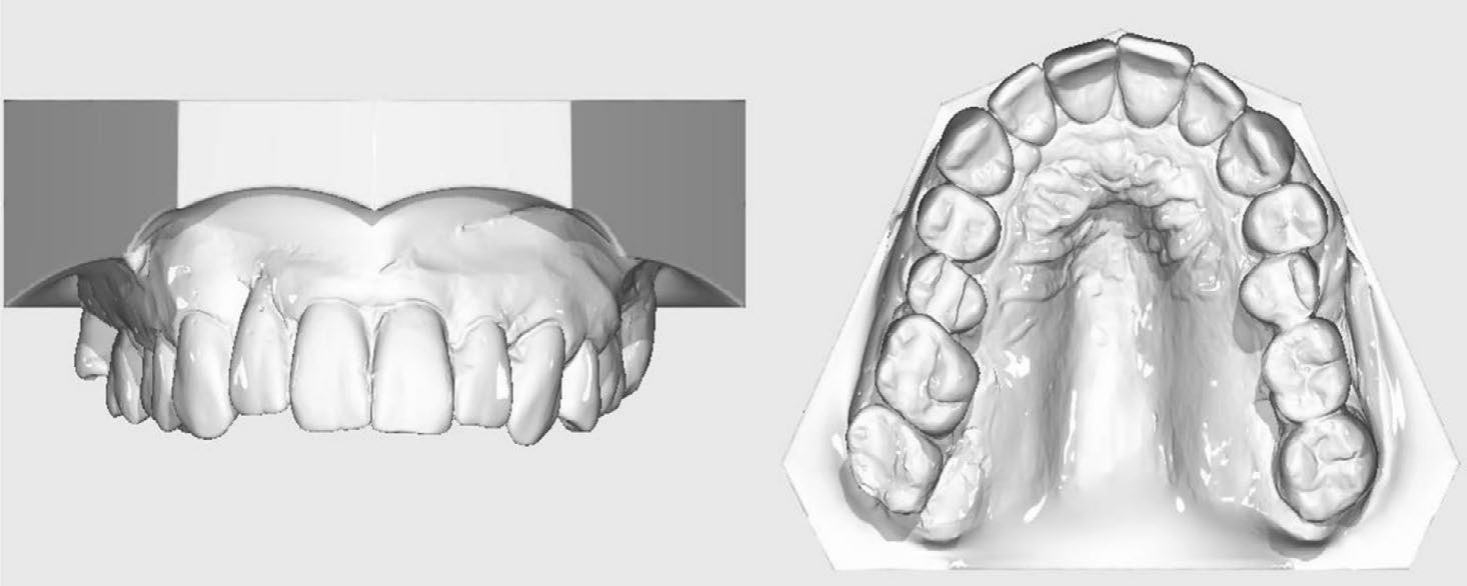
Maxillary model after completion of therapy.

After completion of the periodontal pretreatment and the 6‐month reevaluation and stabilization phase (Figure 1), skeletal anchorage was inserted in September 2016 using vestibular mini‐screws in regions 13/14 and 23/24. Furthermore, a maxillary multibracket appliance (22‐in. system) was bonded (teeth 13–23), in combination with a 0.014″ NiTi archwire and bilateral power chains to initiate the intrusion of the upper anterior teeth (Figure 9). Three months later, a bracket was bonded on tooth 25 (Figure 10), and a 0.018″ stainless steel archwire with an intrusion step was inserted. In January 2017, an 18 × 25 stainless steel sectional archwire including an individual loop on tooth 25 was placed. Throughout the next 2 months, intrusion was continued and reinforced especially in region 12. Furthermore, the final alignment of the teeth was improved. The multibracket appliance was removed in June 2017 after 10 months of active treatment (Figures 11 and 12), and a vacuum‐formed retainer was inserted, followed by a bonded retainer (13–23).

## 3 Results

**FIGURE 13:**
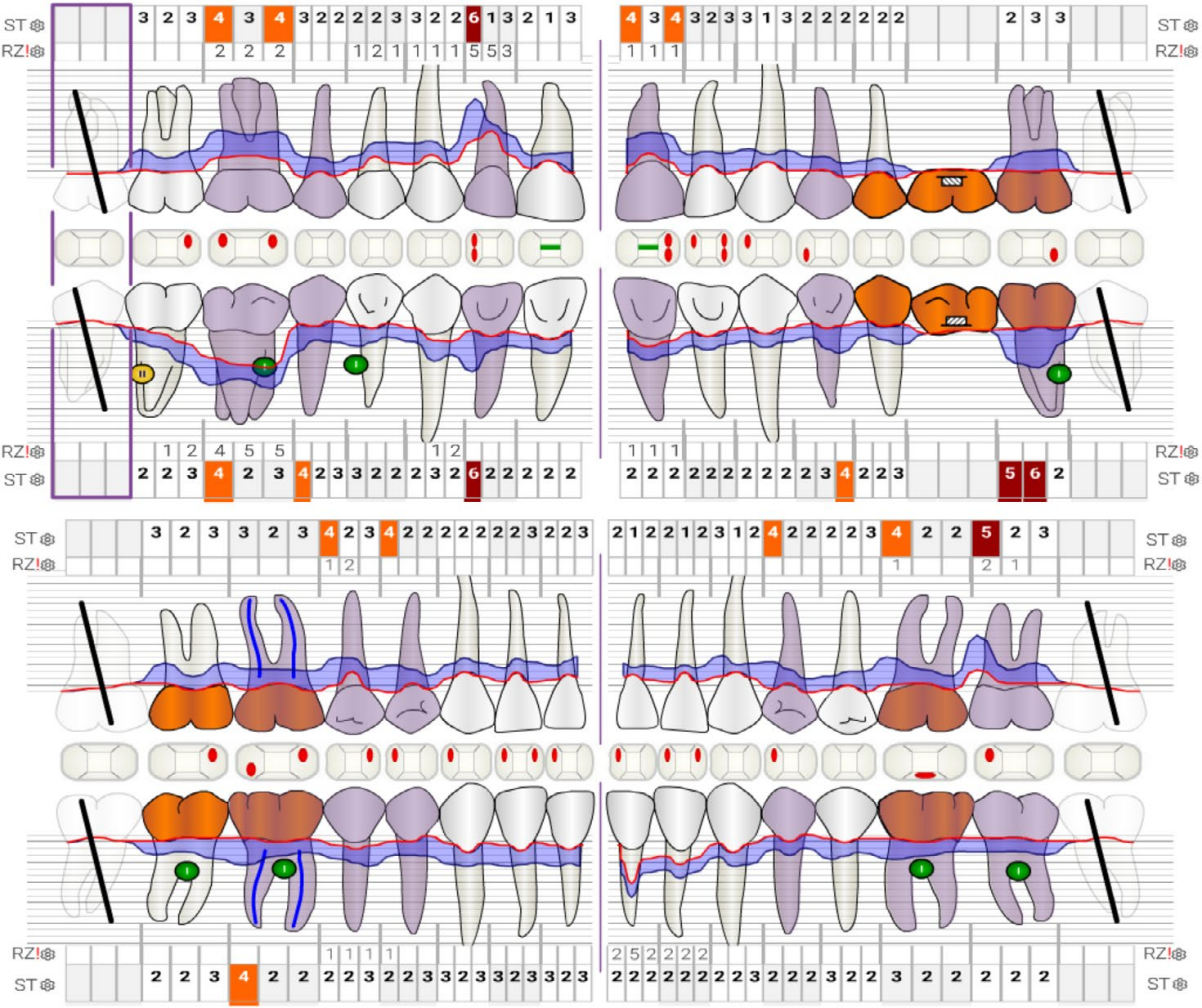
Periodontal status after therapy. Periodontal status after therapy, with RZ indicating gingival recessions and ST indicating probing depths in millimeters.

The final outcome showed successful intrusion and retraction of the protruded and elongated anterior teeth. Tooth 21 was repositioned within the arch, while the lateral diastema was fully closed. Furthermore, the anterior crowding was resolved, and the overjet and overbite were reduced. Posterior occlusion remained unchanged, and the treatment goals were achieved without periodontal compromise. Despite the reduced bone support and advanced age, controlled and stable tooth movement was accomplished through skeletal anchorage. The patient regained lip competence and was highly satisfied with both functional and aesthetic results (Figure 2). To optimize incisal morphology, a final restorative build‐up of the anterior maxillary teeth was planned. Compared with the initial periodontal status (probing depths of up to 9 mm), post‐treatment evaluation showed a substantial reduction in probing depths, with predominantly physiological values and only isolated teeth with probing depths of 6 mm (Figure 13).

## 4 Discussion

The case report highlights the relevance of orthodontic treatment in periodontally diseased patients as part of an interdisciplinary therapeutic concept. The patient exhibited pathologic tooth migration due to advanced periodontitis, particularly in the form of elongation and proclination of the upper front teeth. These changes, along with an increased overjet and overbite, are typical clinical manifestations of severe periodontitis as reported in previous studies [4, 5, 43, 44].

In cases of severe periodontal destruction, imbalances in soft tissue forces can promote further migration of the anterior teeth, whereas in periodontally healthy individuals these forces are usually counterbalanced by the intact periodontium [4, 43]. As described in multiple studies, the intrusion and retrusion of the anterior teeth are particularly important to reposition the incisors to their natural position in these cases [33, 34, 35].

There is evidence of significant correlations between changes in anterior tooth position and lip morphology, whereby greater protrusion of the incisors results in the lips moving forward and upward [37, 38]. Such tooth position changes directly influence the perioral soft tissue and facial profile. Especially in patients with pronounced anterior migration, changes in the facial profile and lip closure are common before treatment [45]. In this context, it would be interesting to further investigate how intrusion and retrusion of the anterior segment affect the lip profile post‐therapeutically in these patients.

Furthermore, the occlusal plane plays a critical role in orthodontic diagnosis and treatment planning, as it influences mandibular rotation and the overall facial profile [46]. In this regard, the FAOP can be used as a valuable reference as previously mentioned [36]. Consideration of the FAOP can help achieve a balanced dentofacial relationship and prevent not only unfavorable skeletal but also soft tissue effects. Nevertheless, the literature provides limited data on how exactly the FAOP influences functional benefits, which would be an interesting aspect for further studies.

In addition to the hard‐tissue improvements, a detailed assessment of the pre‐ and post‐treatment intraoral photographs revealed notable improvements in the periodontal soft tissues. Gingival contour became more harmonious and interdental papillae appeared fuller. These improvements are likely attributable to the combination of prior periodontal stabilization, improved oral hygiene, and the use of controlled orthodontic mechanics that minimized trauma to the periodontium. This observation underscores that orthodontic interventions in periodontally compromised patients can be compatible with, and even supportive of, soft tissue health, provided interdisciplinary planning and carefully controlled biomechanics are employed.

A further key aspect was therefore the close interdisciplinary collaboration between orthodontics and periodontology. The current S3 guideline by the EFP emphasizes that, in patients with stage IV periodontitis, orthodontic therapy can be an integral component of functional rehabilitation, assuming a stable periodontal condition [6]. Therefore, orthodontic treatment started only after successful periodontal therapy.

The limited anchorage capacity of the supporting tissues as well as the increased risk of iatrogenic damage describe the primary challenges in treating periodontally diseased patients.

Similar strategies have been described in the literature, such as Meyer‐Marcotty [30], who emphasized the use of localized segmental mechanics and constant intrusive forces to stabilize teeth in periodontally compromised dentitions.

To address this, segmental appliances and skeletal anchorage elements were also utilized, allowing controlled, low‐force tooth movements while avoiding additional periodontal stress [29, 30]. The case described above demonstrates the benefit of mini screws for targeted intrusion and stabilization of mobile and elongated anterior teeth.

Hong et al. [47] also highlighted the use of segmental archwires and an orthodontic force system based on the centre of resistance to preserve periodontal support in a middle‐aged patient with severe alveolar bone loss.

The resulting improvement in the vertical position of the anterior teeth led to functional and aesthetic benefits, particularly regarding long‐term tooth preservation.

Similar positive outcomes have been reported in other interdisciplinary case series and long‐term follow‐ups. Cardaropoli et al. [48] demonstrated successful management of intrabony defects in stage IV periodontitis with combined periodontal‐orthodontic therapy, achieving aesthetic and functional improvements. Likewise, Cao et al. [49] reported stable results over a 10‐year follow‐up after multidisciplinary treatment of severe periodontitis, reinforcing the potential for long‐term stability when orthodontic interventions are carefully coordinated with periodontal therapy.

A key difference to orthodontic treatment in periodontally healthy patients is the even more important role of retention. Due to reduced periodontal support and an increased tendency for relapse, long‐term retention is indicated [1]. In this context, the use of a retainer and a centric splint is exemplary. Furthermore, the application of light, precisely dosed forces is critical in periodontally diseased patients to prevent further damage and to ensure controlled movement [1, 29]. Therefore, segmental archwires are preferred, allowing low, intrusive forces on isolated tooth groups [30].

The case focused only on the maxillary arch, meaning that sagittal or transverse discrepancies in the mandible could not be fully corrected. This reflects a treatment prioritization that often targets the correction of pathologic migration in the maxilla, particularly the upper front teeth [30].

### 4.1 Outcome Assessment

Treatment success was evaluated based on clinical findings. Reduction of overjet and overbite, correction of anterior tooth position, and closure of interdental spaces were achieved. Periodontal maintenance records during and after orthodontic therapy showed stable probing pocket depths and no signs of increased inflammation.

The patient reported a clear subjective improvement in oral function and aesthetics. In addition, the patient described improved confidence in social interactions and a perceived enhancement of overall oral comfort.

Functionally, normalization of anterior guidance and reduction of traumatic incisal contacts were achieved. The elimination of lip entrapment resulted in improved lip closure and more stable anterior contact relationships.

At follow‐up, the case demonstrated stable occlusal and periodontal conditions under supportive periodontal therapy and retention. The achieved tooth positions remained unchanged, and no relapse of pathological tooth migration was observed.

### 4.2 Limitations and Strengths

As inherent to case reports, the findings are based on two individual cases and cannot be generalized. No standardized patient‐reported outcome measures were applied, and subjective improvements were assessed qualitatively.

No post‐treatment lateral cephalometric radiographs were obtained; therefore, a quantitative cephalometric analysis and superimposition of skeletal and dentoalveolar changes were not performed. Changes in incisor inclination and soft tissue profile were evaluated clinically and photographically.

Despite these limitations, the presented approach demonstrates important strengths. Orthodontic treatment was initiated only after documented periodontal stabilization and performed using segmented mechanics with skeletal anchorage to minimize reactive forces. The therapeutic focus was deliberately restricted to correction of pathological anterior migration, prioritizing periodontal preservation. Furthermore, treatment planning incorporated soft tissue considerations and lip‐incisor interaction using the FAOP as a functional‐aesthetic reference.

## 5 Conclusion

This case report illustrates that the correction of pathological tooth migration is not only achievable but effective in patients in later adulthood, demonstrating that age alone is not a barrier to orthodontic treatment in periodontally diseased patients. The application of light, controlled forces as well as particularly intrusive movements allows for safe and predictable tooth movement while preserving periodontal health. Additionally, the FAOP serves as a useful reference for guiding occlusal and aesthetic outcomes, but the key to success lies in interdisciplinary planning and periodontal stabilization.

## Author Contributions

**Lisa‐Marie Mai:** conceptualization, writing – original draft, writing – review and editing. **Bernhard Wiechens:** writing – review and editing. **Anja Quast:** writing – review and editing. **Gaayathiri Suntharalingam:** investigation, resources. **Valentina Hrasky:** investigation, resources. **Philipp Meyer‐Marcotty:** conceptualization, investigation, resources, writing – review and editing.

## Funding

The authors have nothing to report.

## Ethics Statement

The authors have nothing to report.

## Consent

The authors confirm that written informed consent was obtained from the patient for publication of this case report and accompanying images.

## Conflicts of Interest

The authors declare no conflicts of interest.

## Data Availability

Due to privacy and ethical restrictions, patient data are not publicly available. Further details are available from the corresponding author upon reasonable request.
